# Fine ash from the Campanian Ignimbrite super-eruption, ~ 40 ka, southern Italy: implications for dispersal mechanisms and health hazard

**DOI:** 10.1038/s41598-025-01100-4

**Published:** 2025-06-10

**Authors:** Flaminia Gianchiglia, Paolo Ballirano, Biagio Giaccio, Andreas Koutsodendris, Sebastien Nomade, Alessandro Pacella, Danilo M. Palladino, Jörg Pross, Daniel Veres, Gianluca Sottili

**Affiliations:** 1 Department of Earth Sciences , Sapienza University of Rome, Rome, Italy; 2 Institute of Environmental Geology and Geoengineering, CNR, Rome, Italy; 3 Institute of Earth Sciences, Heidelberg University, Heidelberg, Germany; 4 Laboratoire des Sciences du Climat et de l’Environnement, UMR 8212, CEA-CNRS, CEA-UVSQ, University of Paris-Saclay, Gif sur Yvette, France; 5 Institute of Speleology, Romanian Academy, Cluj-Napoca, Romania

**Keywords:** Super-eruption, Fine ash, Campanian Ignimbrite, Terminal fall velocity, Health hazard, Aerodynamic properties, Natural hazards, Solid Earth sciences

## Abstract

Super-eruptions disperse volcanic ash over vast areas, impacting the environment and human health. Fine ash, particularly its respirable fraction (< 4 µm), poses a significant health hazard by inhalation due to its high dispersal potential. Understanding the aerodynamic properties but also composition of ash particles is fundamental to constrain dispersal and deposition mechanisms in both proximal and distal environments. Current atmospheric dispersal models rely on empirical drag equations calibrated with geometric shape descriptors. However, these models often overlook the effects of the actual particle density, as a uniform componentry is typically assumed. In addition, particles have variable shapes but such data from super-eruptions remains limited and no standardized measurement methods exist. Here, we determine the terminal fall velocity (*v*_*t*_) of fine ash from the Campanian Ignimbrite super-eruption (~ 40 ka, Campi Flegrei), by evaluating the components and particle shapes from proximal to ultra-distal locations. To verify the attribution of the proximal sample to the CI eruption, a ^40^Ar/^39^Ar dating was performed, allowing its correlation with the ultra-distal deposits. Results show that, due to the influence of shape and density, glass particles exhibit lower *v*_*t*_ compared to mineral phases (*v*_*t*, feldspar_/*v*_*t*, glass_ = 1.05 ± 0.03, *v*_*t*, SiO2_/*v*_*t*, glass_ = 1.09 ± 0.02), enabling greater travel distances. Drag equations accounting for measured particle shapes differ significantly from spherical approximations. The spherical model overestimation of *v*_*t*_ highlights the necessity of shape-specific models to produce more accurate dispersal predictions. Extremely low *v*_*t*_ (< 0.1 cm/s) for respirable ash fraction, which indicates prolonged atmospheric suspension and long-time resuspension potential, along with the presence of cristobalite, lead to important implications for health hazards. These findings further enhance our understanding of volcanic ash aerodynamic behaviour and the far-reaching impact of super-eruptions**.**

**Supplementary Information:**

The online version contains supplementary material available at 10.1038/s41598-025-01100-4.

## Introduction

Volcanic ashes from large explosive eruptions are extremely impactful for both the abiotic and biotic component of the ecosystems due to the wide areas that can be affected^1^. Indeed, its dispersal and deposition, especially during super-eruption events, defined as large-scale, caldera and ignimbrite-forming explosive eruptions with very high intensity (magma flux, > 10^10^ kg/s), can have consequences for global-scale climate, ecosystems, and atmospheric processes^2–4^. Volcanic ash can also impact aviation such as plane’s engines^5^ and represent a source of water contamination^6^ and lastly but not the least have great potential health hazard by inhalation^7^. The effects of volcanic ash can also persist for decades due to their resuspension in the environment by wind or anthropogenic activities, exacerbating their primary impact^8,9^. Understanding the mechanisms of volcanic ash transport and deposition is therefore a fundamental first step to assess their multiple impact.

The fractionation and transport of ash particles are strongly controlled by their aerodynamic properties^10–12^ which, in turn, are strongly influenced by their modal composition, grain size, and shape^13^. These features determine the terminal fall velocity of volcanic ash particles which, along with meteorological conditions at the time of the eruption, controls primally the residence time of the particles into the atmosphere^14^. Volcanic ash with sizes of < 200 μm has atmospheric residence time on the order of 1 h or more^15^, and it was predicted that particles of < 10 μm in size have residence time > 10 days^16^, thus being easily transported to greater distances from the source.

Numerical atmospheric dispersal models allow for the identification of the area potentially affected by volcanic ash fallout; the calculation of the terminal fall velocity is crucial for the development of these models and can be calculated through the following equation:

1 $$v_{t} = \sqrt {\frac{{4gd_{eq} \left( {\rho_{p} - \rho_{f} } \right)}}{{3\rho_{f} C_{D} }}}$$

where *g* is the gravitational acceleration, *d*_*eq*_ is the diameter of the volume-equivalent sphere, *ρ*_*p*_ and *ρ*_*f*_ are particle and fluid density, respectively, and *C*_*D*_ is the drag coefficient.

An estimation for non-spherical irregular particles yields values of *v*_*t*_ < 0.26 m/s for fine ash (< 63 μm), 0.16–11.9 m/s for coarse ash (63 μm–2 mm) and 5.9–57.4 m/s for lapilli (2–64 mm)^11^, from deposits of various volcanic eruptions from mafic (Kilauea^17^, Masaya Fontana Lapilli^18^, Villarrica^19^, Stromboli^20^), intermediate (Cotopaxi^21^, Llaima^22^), and silicic^23^ eruptions and volcanic ash from Equatorial Pacific cores and a sub-aerial tuff sequence on San Miguel^24^.

In this above-mentioned equation, the drag coefficient is a function of different parameters such as Reynolds number and shape descriptors of a particle. The Reynolds number is defined as:

2 $$Re = \frac{{\rho_{f} v_{t} d_{eq} }}{\mu }$$

where *ρ*_*f*_ is the fluid density, *d*_*eq*_ is the diameter of the volume-equivalent sphere and *μ* is the fluid dynamic viscosity.

Many models have been proposed to calculate the drag coefficient (﻿*C*_*D*_); some of them approximate particles to spheres^25,26^ whereas other ones consider the shape of irregular particles^10,12,24,27–34^. These latter were derived both empirically and experimentally, through vertical tunnel experiments, and provide terminal velocity values with better accuracy than models considering spherical-approximated particles. Many studies have been carried out over the past decades to quantify the shape of irregular particles^15,35–45^. To date, however, there is no standardized method for measuring shape. Indeed, the most appropriate shape descriptor depends on several factors such as the texture of the particles and the morphological analyses and the aim of the research. Recent developments in analytical techniques have facilitated the analysis of 3D shapes; the most common shape descriptors used in drag equations are *sphericity *(*ψ*)*,*^12,30,31^, which, however, is defined differently depending on the model considered, as well as *flatness *(*f*) and *elongation *(*e*) parameters^10,43^. In addition, some atmospheric dispersal models still assume the same composition and density for all particles, thus ignoring the effects of different components and aerodynamic properties, leading to an inaccurate estimate of the ash dispersal area. In this context, the determination of the shape through shape descriptors, along with component identification, is therefore of crucial importance.

The reconstruction of componentry, aerodynamics properties, dispersal mechanisms, and mobility of fine ash from super-eruptions has important implications because:

1. The finer the particles are, the greater their atmospheric residence time and dispersal area^16,46,47^. Finest ash can travel up to thousands km away from the volcanic vent^16^ and can be remobilized for years^8^.
2. The finest particles represent a hazard to human health. Indeed, the < 10 μm fraction (PM_10_), known as the *thoracic* fraction, can reach the bronchial tubes, whereas the < 4 μm fraction, the *respirable* one, can reach the alveoli, where chronic respiratory diseases may develop^7^.

However, distal and ultra-distal ash deposits, mainly consisting of fine ash, are often not preserved as coherent layers due to their thinness and high potential for post-depositional remobilization and alteration. As a result, far-travelled fine ash is frequently not included in the isopach maps limits of past eruptions, leading to an inaccurate estimation of the transported ash mass for example^48^, and aerodynamic data for super-eruptions are therefore limited.

The determination of the mineralogical composition of volcanic ash also plays a key role in assessing their impact on human health. Indeed, the presence of specific minerals, such as cristobalite, poses a potential health risk by inhalation^7^. Identifying potential hazardous mineral phases can contribute to a better risk assessment.

On these grounds, the mineralogical characterization of volcanic ashes and the determination of their aerodynamic characteristics are crucial to model the dispersal area and impact of volcanic plumes from large explosive events. The determination of these parameters from both proximal and distal fine ash of super-eruptions can provide a better estimation of their dispersal potential, with an improvement of eruption parameter estimates (e.g., magnitude and intensity^48^) and eruptive style^44,49^.

**Fig. 1:**
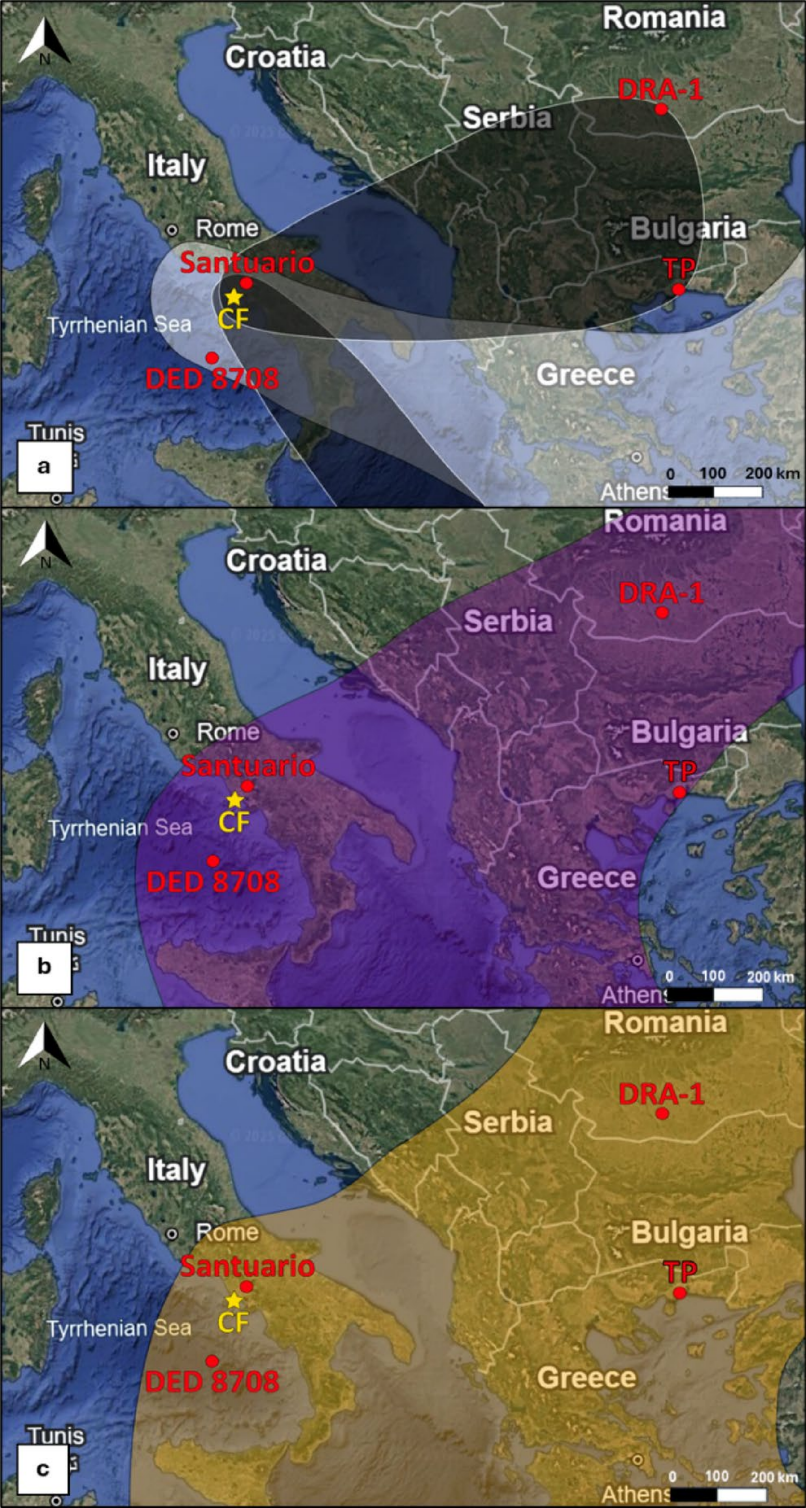
Proximal, middle, distal, and ultra-distal sampling locations of the investigated samples (red dots). Yellow star: approximate source of the CI eruption (CF: Campi Flegrei). (**a**) Dispersal of Plinian and LPF co-PDC (black field) and UPFU co-PDC (white fields) plumes from Smith et al.^60^. Dispersal areal of the (**b**) Plinian and (**c**) co-PDC phases within the 0.1 cm isopach of Marti et al.^58^ model.

Here, we characterize Campanian Ignimbrite (CI) super-eruption pyroclastic products from proximal (Maddaloni site, ~ 33 km from the source), mid-distal (Tyrrhenian Sea site, ~ 200 km from the source, and ultra-distal locations (Tenaghi Philippon and Draganesti-Olt site, ~ 848 km and up to ﻿~ 940 km from the source, respectively) (Fig. 1) by considering different depositional environments (subaerial, marine and lacustrine). Specifically, we determine the componentry and shape of different size ranges of fine ash (i.e., < 4 μm, 5–8 μm, 9–16 μm, 17–63 μm), thus also including the respirable fractions. We perform component analyses to investigate the presence of potential hazardous minerals and to identify changes in the abundance of mineral phases within the different size fractions as a function of the distance from the source. In addition, shape characterization allows us to calculate the terminal fall velocity of single particles. Our goal is to provide new insights into fractionation, transport, and resuspension mechanisms of volcanic fine ash, with implications for environmental impact and health hazard assessment of super-eruptions.

## Campanian Ignimbrite eruption

The 39.85 ± 0.14 ka^50^ Campanian Ignimbrite (CI) super-eruption of Campi Flegrei Volcanic District (Naples, Southern Italy) is the largest known volcanic event of the past 200 ka in Europe^51,52^. This caldera-forming eruption may have caused “volcanic winter” in Europe^53^ which, combined with stadial conditions during Heinrich Event 4 of the Last Glacial, may have impacted early Upper Palaeolithic populations^54,55^, although it failed to have lasting consequences for Neanderthals or early modern humans^56^. The products of the Campi Flegrei belong to the alkali-potassic series, specifically low-potassium terms (LKS) dominate. As a result, the primary paragenesis minerals include clinopyroxenes, plagioclase, olivine, and sanidine.

The CI eruption had two main explosive phases:

1. An early Plinian phase that produced a sustained Plinian column up to 44 km high^57,58^, which deposited a total volume of 54 km^3^ of tephra (~ 23 km^3^ DRE)^58^ to the east and south (Fig. 1), encompassing an area of ~ 1.3 million km^2^^58–60^.
2. A pyroclastic current phase. This phase is characterized by turbulent pyroclastic density currents (PDCs) with an average runout distance of ~ 75–80 km^61,62^ and a mass flow rate of ~ 10^11^ kg/s which overcame reliefs higher than 1000 m^63^, covering an on-land area of ~ 6000 km^2^^62^. The PDCs resulted in co-ignimbrite plumes that reached 37 km in height^58^ and dispersed ashes up to 3200 km away from the vent^60,64^ over an area of ∼3 million km^2^, producing ~ 154 km^3^ (~ 62 km^3^ DRE) of deposits^58^.

The CI deposited therefore an estimate total of 208 km^3^ of pyroclastic material (155–235 km^3^ DRE)^58^, generating volcaniclastic sequences in the proximal areas (∼80 km) where both Plinian fall deposits and pyroclastic currents and co-ignimbritic products can be clearly separated, with from bottom to top^65–67^: (1) the Plinian pumice fallout (PPF); (2) unconsolidated stratified ash flow (USAF); (3) a welded grey ignimbrite (known as ‘Piperno’ in the most proximal area and as WGI in the less proximal one); (4) a lower pumice flow unit (LPFU), and (5) a coarse lithic breccia unit (BU) interbedded with a welded spatter unit (SU), which with distance become a lithified yellow tuff (LYT); (6) the upper pumice flow unit (UPFU). The lithic breccia deposits (collectively known as Breccia Museo) were deposited during the caldera collapse phase^68,69^, thus stratigraphically delimiting the pre- and post-caldera formation.

From 80 km from the vent onwards, pyroclastic current deposits are no longer present, and at medial distances (up to ~ 600 km) the Plinian deposit is overlain by the co-ignimbrite tephra separated by a sharp contact. In the even more distal deposits (up to 850 km), on the other hand, the two phases began to be mixed in the atmosphere and are no longer identifiable individually^70^. However, it is still possible to determine the presence of both phases because of the two different grain-size modes (co-ignimbrite ashes have finer grain sizes than the Plinian ones). With further distance (over 850 km) the deposits are unimodal, as the tephra from the two phases were completely mixed in the atmosphere and the two phases can no longer be recognizable^70^. Marti et al.^58^ calculated that the co-ignimbrite phase represented almost 74% of the total bulk volume for the eruption. Engwell et al.^70^ determined, through grain-size data that, within a radius of 850 km from the source, the 40 ± 5% of the deposits volume is represented by the Plinian phase of the eruption and 60 ± 6% is from the co-PDC phase. Smith et al.^60^ were able to differentiate, through glass composition analyses, the Plinian and the co-PDC phase pre-caldera collapse (LPFU), with the most evolved composition, versus the co-PDC phase during/after the caldera collapse (UPFU), with the least evolved composition. The discrimination of the two components was performed using the K_2_O content, as the Plinian phase shows a narrower range and a lower content in K_2_O (7.02–7.53 wt%/, whereas the UPFU has a higher content of K_2_O and a wider range (9.45–10.26 wt%). Smith et al.^60^ also shown that in ultra-distal locations (over 1000 km) northeast and southeast of Campi Flegrei, more than 94% of the fallout volume is associated with the late co-PDC phase (UPFU). Considering the wide dispersal area and the distances reached by the tephra of the CI, this super-eruption represents an ideal candidate to assess the dispersal potential of fine ash and its impact on the environment and for humans.

## Results

^40^Ar/^39^Ar dating of the proximal Santuario sample allowed to confirm its belonging to the CI eruption, thereby enabling its correlation with the other more distal samples. The dating results are presented as probability diagrams in Supplementary Fig. S1. The ages of 17 individual crystals ranged from 38.7 ± 1.8 ka (2σ) up to 107.9 ± 1.4 ka (2σ). The resulting probability diagram displays two main modes (Supplementary Fig. S1), with the youngest including the largest number of date crystals (c.a. 13 crystals). This youngest population allows the calculation of a weighted mean age of 39.88 ± 0.45 ka (2σ, MSWD = 0.30, *P* = 1) (Supplementary Table S1 and S2), despite the presence of xenocrystals we interpret this age of the youngest population as the eruption age of this sample, which is indistinguishable from that of the CI (39.9 ± 0.1 ka).

Concerning the ash componentry, X-Ray Powder Diffraction (XRPD, Supplementary Fig. S2) analyses performed on each sample revealed the presence of different mineral phases. We identify abundant feldspar and quartz occurring in all samples, as well as calcite, analcite, illite, kaolinite, pyroxenes, cristobalite, hematite, halite, and gypsum.

Overall, the quantification analyses of the glass, vesicle, and crystals in the top, middle, and base Santuario sub-samples show minor glass and crystal content variations across the layer. Vesicles are the dominant element in all three sub-samples, consistently accounting for more than 76% of the area (Supplementary Fig. S3a) i.e., within the range of 72–84% reported for the “basal pumice-rich facies” for medial deposits of the CI, located from 30 to 79 km from the vent^71^. Glass is the second most abundant component, representing 24% of the area in the top sub-sample and slightly decreasing to ~ 20% in the middle and the base. Crystals are the least abundant phase in the Santuario sample, with no crystals occurring at the top, 0.3% of crystals in the middle, and a slight increase to 1.1% at the base (Supplementary Fig. S3a).

The quantification of glass, feldspar, and crystalline SiO_2_ in the fine fraction (< 63 µm) of the more distal samples (DED 8708, TP, and DRA-1) reveals distinct trends. The SEM analysis does not allow discrimination between cristobalite and quartz, but only the identification of a SiO_2_ mineral phase, which is therefore reported in the quantification as crystalline SiO_2_. Discrimination of sanidine, albite and plagioclase was instead possible, but they were grouped as feldspars. In the DED8708 sample, there is a gradual increase in the glass amount as particle size increases, feldspars remain constant in all particle size fractions, whereas crystalline silica content decreases with increasing grain size fraction, being most abundant in < 4 µm fraction (Supplementary Fig. S3b). In the TP sample, glass is mainly concentrated in the intermediate fraction (5–8 µm), indeed feldspar’s contribution is greater in finer (< 4 µm) and coarser (9–63 µm) fractions, with crystalline SiO_2_ representing only a marginal portion of these latter (Supplementary Fig. S3c). In the DRA-1 sample, similarly to the DED 8708 sample, the glass portion increases with particle size, corresponding to a decrease in the feldspar content. By comparing the samples, glass is the dominant phase, whereas feldspar and crystalline SiO₂ are present in smaller amounts. DED 8708 sample contains higher amounts of feldspar and crystalline SiO_2_ in all particle size fractions than the others, with crystalline SiO_2_ being absent in the DRA-1 sample. In contrast, the glass portion increases in more distal samples (TP and DRA-1) (Supplementary Fig. S3b–d).

The grain size distribution of the mid-distal samples (Table 1, Supplementary Fig. S4) shows that all samples have a predominance of coarser particles. In DED 8708 (Tyrrhenian Sea), the mid-distal sample, the coarser fraction (63 µm – mm size) represents the most significant portion of the sample, indicating the presence of coarser material nearer the source (Supplementary Fig. S4). By contrast, the ultra-distal samples, TP (Tenaghi Philippon, Greece) and DRA-1 (Romania), exhibit a shift towards finer fractions (17–63 µm and 9–16 µm), which increase in abundance. The observed trend reflects the progressive settling of larger particles nearer the source and the transportation of finer particles over longer distances.

**Table 1:** Weight percentage (wt%) distribution of grain size fractions for each sample.

	DED 8708	TP	DRA-1
< 4 µm (wt%)	3	2	1
5–8 µm (wt%)	3	2	2
9–16 µm (wt%)	3	6	6
17–63 µm (wt%)	28	51	43
63 µm-mm size (wt%)	64	38	48

**Fig. 2:**
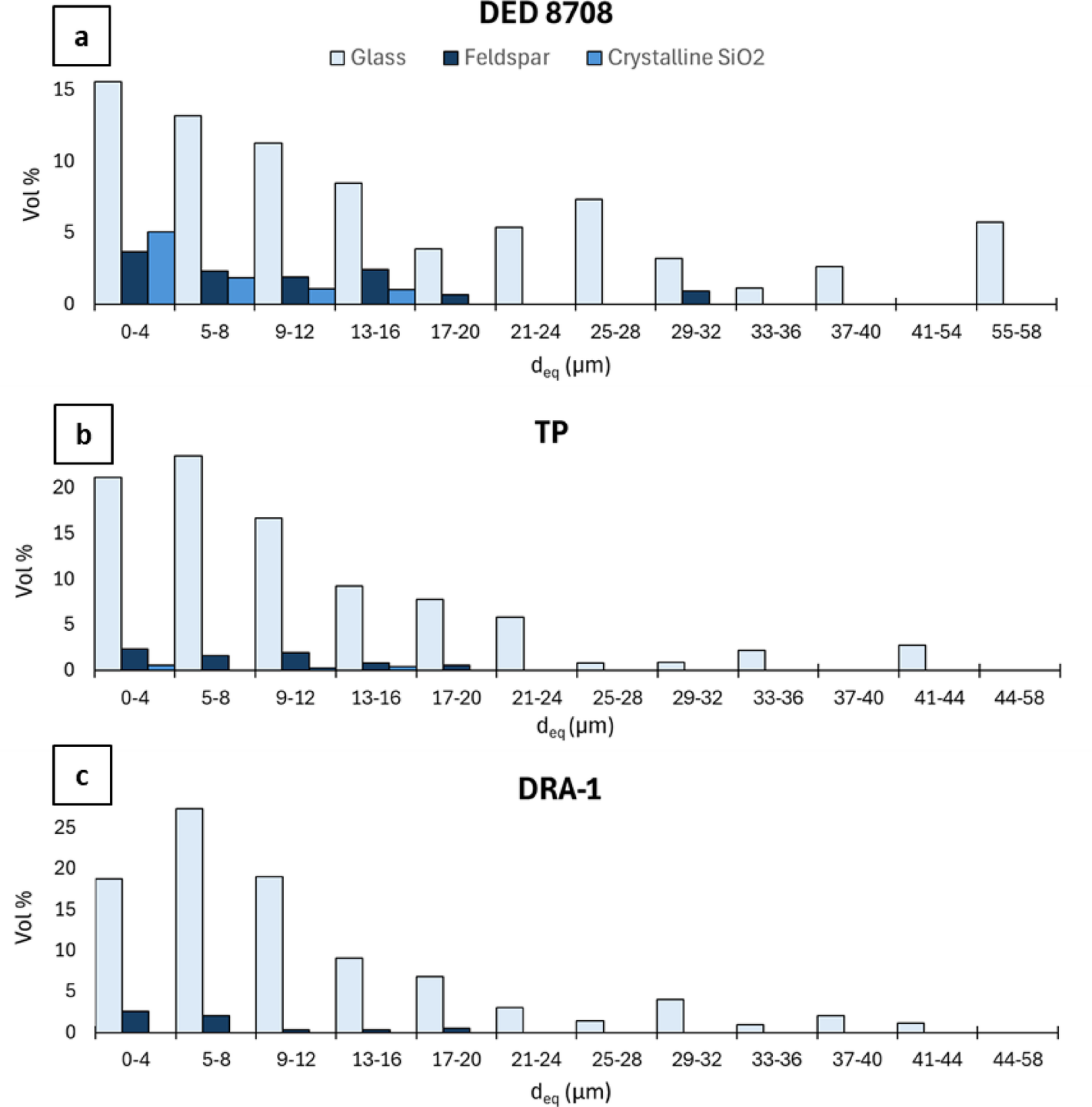
Histograms of cumulative vol% vs equivalent diameter, showing the total grain size distribution of glass, feldspar, and crystalline SiO_2_ components as a function of particle size in DED 8708 (**a**), TP (**b**), and DRA-1 (**c**) samples. The mean percentage error is below 1 vol%. Average density of components: *ρ*_*glass*_ = 2500 kg/m^3^, *ρ*_*feldspar*_ = 2560 kg/m^3^, *ρ*_*SiO2*_ = 2650 kg/m^3^).

Figure 2 illustrates the total grain size distribution of each component in the fine fraction (< 63 µm) of the mid-distal samples. In the DED 8708 (Tyrrhenian) sample, glass particles reach sizes of up to 57 µm, in comparison feldspars and crystalline SiO_2_ have a maximum size of 30 µm and 16 µm, respectively, being mainly very fine in size (Fig. 2a). Similarly, in the TP and DRA-1 samples, glass particles reach sizes up to 44 µm and 43 µm and the feldspars reach a maximum size of 17 µm and 18 µm, respectively. Crystalline SiO_2_ reaches a maximum size of 13 µm in the TP sample, whereas it is absent in the DRA-1 sample (Fig. 2b,c). Overall, in all samples, there is a general concentration of mineral phases (feldspars and SiO_2_) in the smaller sizes, compared to glass which covers a wider size range, reaching larger dimensions. Furthermore, the maximum sizes of both glass and mineral phases decrease from the most proximal sample (DED 8708) to the most distal samples (TP and DRA) (Fig. 2).

**Fig. 3:**
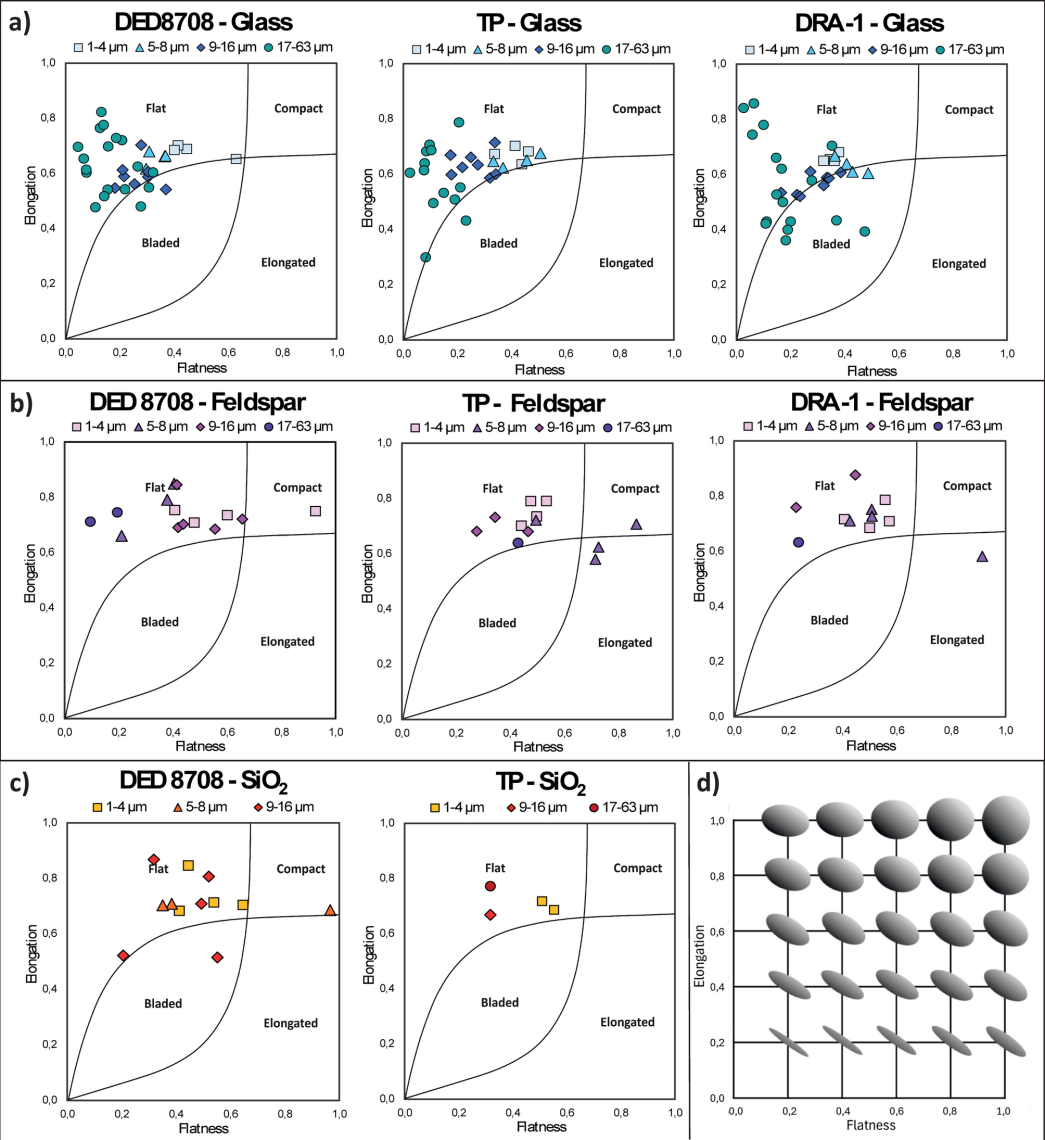
Particle shape classification based on the classification system proposed by Angelidakis et al.^72^. (**a**) glass, (**b**) felspar, and (**c**) crystalline SiO_2_ components for each sample. (**d**) Particle shapes diagram modified by Maramizonouz & Nadimi^73^.

The morphology of the particles of each component is presented in Fig. 3 following the classification system proposed by Angelidakis et al. 2021^72^. The values of the shape descriptors measured are given in Table 2. A similar trend is observed for the glass particles across all samples: most particles of all size fractions have a *flat* shape, with only a few particles resulting in a *bladed* shape. Grains in 17–63 µm fraction are most flattened with various degrees of elongation, as the size decreases (9–16 µm, 5–8 µm, and < 4 µm), particles show a slight reduction in flatness and a more constant elongation, tending towards compact. Feldspars show a smaller elongation range than glass and are mainly *flat* in shape. The variation of flatness in the particle size fractions of feldspars varies between samples; in DED 8708, < 4 µm and 9–16 µm fractions are those which tend to be more compact and less flat compared to the other fractions, in contrast with TP and DRA-1 samples, in which the finer fractions (5–8 µm and < 4 µm) are the less flattened, similarly to the glass. In the TP sample, the 5–8 µm fraction presents the lowest degree of flatness, with a concentration of compact and elongated particles. SiO_2_ particles show a dominant *flat* shape; in the DED 8708 sample, the particle shape does not seem to be influenced by the size, in contrast, the finer fraction (< 4 µm) is less flat. In general, glass exhibits more flat and bladed shapes, especially for the coarser fraction (17–63 µm), with respect to the mineral phases, which tend to be more spherical (Table 2).

**Table 2:** Values of shape parameters (*flatness, elongation* and *sphericity*) for the different grain size fraction (< 4, 5–8, 9–16, 17–63 µm) of each component (glass, feldspar and crystalline SiO_2_) for each sample.

Sample	Component	d_eq_ (µm)	*f *(flatness)	*e *(elongation)	*Ψ *(sphericity)
DED 8708	Glass	< 4	0.4—0.6	0.7	0.7–0.8
		5–8	0.3–0.4	0.6–0.7	0.6
		9–16	0.2–0.4	0.5–0.7	0.4–0.6
		17–63	0.05–0.3	0.5–0.8	0.2–0.6
	Feldspar	< 4	0.4–0.9	0.7–0.8	0.7–0.9
		5–8	0.2–0.4	0.7–0.8	0.5–0.8
		9–16	0.4–0.7	0.7–0.8	0.7–0.9
		17–63	0.1–0.2	0.7	0.3–0.5
	Crystalline SiO_2_	< 4	0.4–0.6	0.7–0.8	0.7–0.8
		5–8	0.4–1	0.7	0.7–0.9
		9–16	0.2–0.6	0.5–0.8	0.5–0.8
TP	Glass	< 4	0.3–0.5	0.6–0.7	0.6–0.7
		5–8	0.3–0.5	0.6–0.7	0.6–0.7
		9–16	0.2–0.3	0.6–0.7	0.4–0.6
		17–63	0.02–0.2	0.3–0.7	0.1–0.5
	Feldspar	< 4	0.4–0.5	0.7–0.8	0.7–0.8
		5–8	0.5–0.9	0.6–0.7	0.8–0.9
		9–16	0.3–0.5	0.7	0.6–0.7
		17–63	0.4	0.6	0.7
	Crystalline SiO2	< 4	0.5–0.6	0.7	0.8
		9–16	0.3	0.7	0.7
		16–63	0.2	0.8	0.6
DRA-1	Glass	< 4	0.3–0.4	0.6–0.7	0.6–0.7
		5–8	0.4–0.5	0.6–0.7	0.6–0.7
		9–16	0.2–0.4	0.5–0.6	0.5–0.6
		17–63	0.03–0.5	0.4–0.9	0.2–0.7
	Feldspar	< 4	0.4–0.6	0.7–0.8	0.7–0.8
		5–8	0–4-0.9	0.6–0.8	0.7–0.9
		9–16	0.2–0.4	0.8–0.9	0.6–0.8
		17–63	0.2	0.6	0.6

**Fig. 4:**
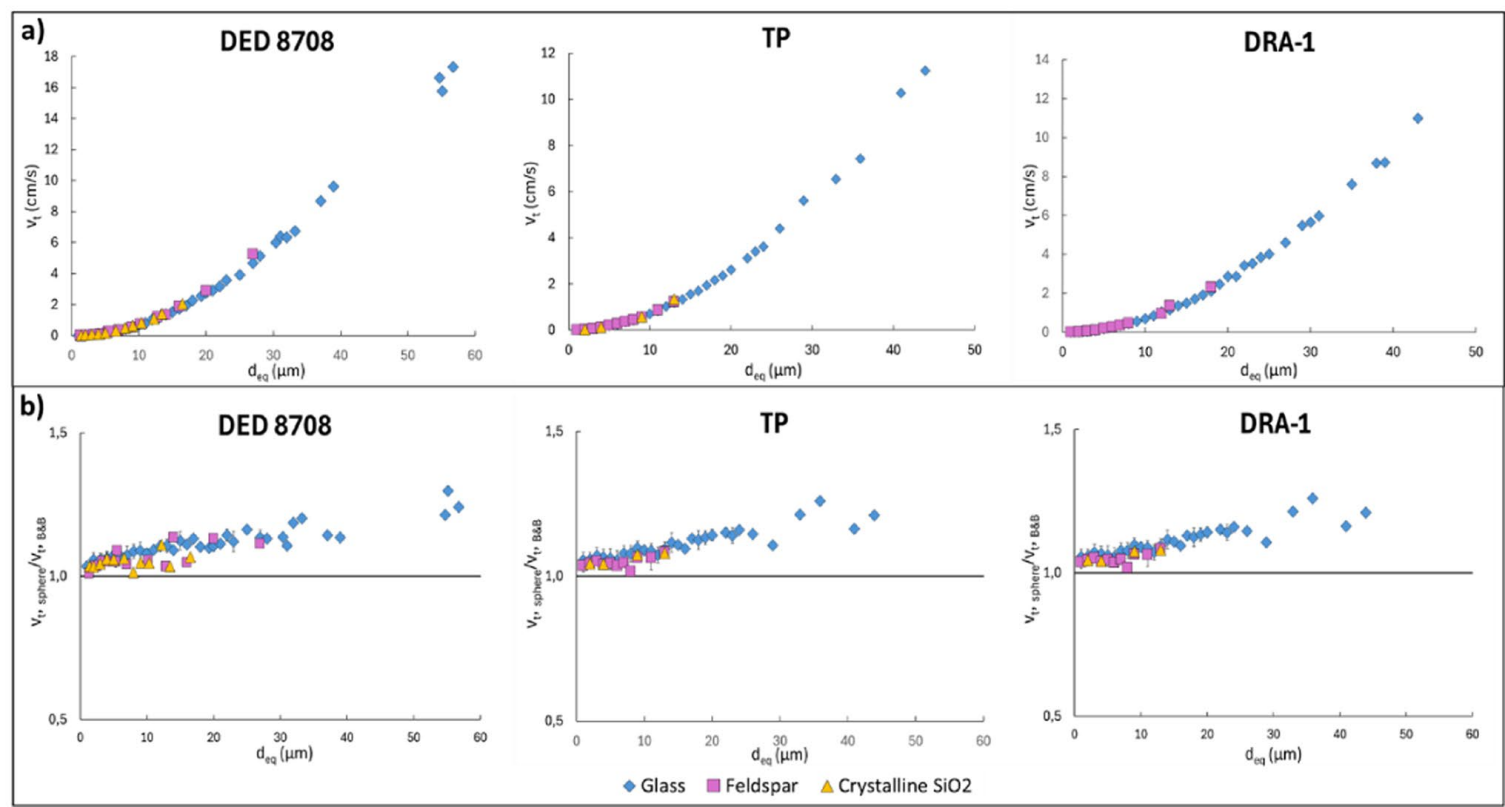
(**a**) Terminal fall velocity calculated through the Bagheri and Bonadonna^10,11^ model and (**b**) *v*_*t,*__*sphere*_/*v*_*t,*__*B&B*_ ratio as a function of the size of the different components. The error bars represent the standard deviation of the mean. Where error bars are not visible, the estimated error is very low (ranging between 0.2 and 0.001) and smaller than the symbol size.

Regarding the terminal fall velocity, in Fig. 4a we can observe the *v*_*t*_, calculated through the shape descriptors (Table 2), as a function of the equivalent diameter, *d*_*eq*_, for volcanic ash particles falling in the atmosphere at sea-level conditions (*ρ*_*f*_ = 1.225 kg/m^3^, *µ* = 1.789 × 10^5^ N s/m^2^). A different particle density is used depending on the component, distinguishing between the different types of feldspars (*ρ*_*glass*_ = 2500 kg/m^3^, *ρ*_*sanidine*_ = 2520 kg/m^3^, *ρ*_*albite*_ = 2620 kg/m^3^, *ρ*_*plagioclase*_ = 2670 kg/m^3^, *ρ*_*SiO2*_ = 2650 kg/m^3^). For all samples the *v*_*t*_^11^ of ash particles of each component follows a general trend, increasing with particle size (*d*_*eq*_). The *v*_*t*_ values result to be very low, in the order of cm/s, up to 18 cm/s for the DED 8708 sample, and up to 11 cm/s for TP and DRA-1 samples. The average terminal velocity of mineral phases is slightly higher (*v*_*t,* minerals/_*v*_*t,* glass_ = 1.07 ± 0.03) than that of glass particles.

Figure 4b illustrates the ratio between the spherical model *v*_*t*_^26^ and the shape model *v*_*t*_ of Bagheri and Bonadonna^10,11^ (*v*_*t,*__*sphere*_/*v*_*t,*__*B&B*_*)* as a function of particle size. The ratio results in all cases greater than 1, particularly for glass, and increases with increasing *d*_*eq*_ for all components.

The average *v*_*t*_ of the mineral phases is slightly higher (*v*_*t,* minerals/_*v*_*t,* glass_ = 1.07 ± 0.03) than that of the glass. Specifically, feldspars exhibit an average *v*_*t*_ of 1.05 ± 0.03 times higher than glass (approximately 5%), while crystalline SiO₂ reaches values about 1.09 ± 0.02 times higher (approximately 9%). Individually analyzing the effects of density and shape on terminal velocity, it is evident that both contribute to the increase in *v*_*t*_  observed in the mineral phases (Supplementary Fig. S5a,b). In particular, considering only the shape and the density influence on *v*_*t*_, respectively, the ratio is *v*_*t, S feldspar*_*/v*_*t,*__*S glass*_ = 1.03 ± 0.2 and *v*_*t, ρ feldspar*_*/v*_*t,*__*ρ glass*_ = 1.02 ± 0.002 for the feldspars, and *v*_*t, S SiO2*_*/v*_*t,*__*S glass*_ = 1.03 ± 0.2 and *v*_*t, ρ SiO2*_*/v*_*t,*__*ρ glass*_ = 1.06 ± 0.001 for the crystalline SiO_2_ (Supplementary Fig. S5c).

## Discussion

This study provides new insights into the aerodynamic behaviour and potential health hazards associated with fine volcanic ash from the CI super-eruption. The relatively high and persistent presence of vesicles in all three proximal sub-samples (top, middle, and base) (Supplementary Fig. S3A) suggests that they were subjected to intense degassing during the eruption, resulting in the formation of a vesicular texture. Concerning the mineral phases identified by XRD, feldspar, and pyroxenes are minerals of primary paragenesis. Pyroxenes were revealed in the TP and DRA-1 samples, whereas in the DED8708 sample the overlapping peaks make the identification uncertain (Supplementary Fig. S2). They are absent in the fine ash fraction (< 63 µm) and are only present in the coarser fractions (> 63 µm). This observation indicates a counter-intuitive fractionation process of pyroxenes, possibly due to the initial crystal size distribution in the fragmenting magma [e.g.,^74^]. Calcite, illite, kaolinite, hematite, and gypsum are interpreted as arising from the secondary alteration processes linked to the depositional environment. Quartz and cristobalite deserve special attention, particularly cristobalite, which poses a potential health hazard in case of prolonged exposure^75^. The products of CI belong to the alkali-potassic series, meaning that quartz and cristobalite are not magmatic mineral phases. The occurrence of quartz and cristobalite in the proximal subaerial sample (Santuario), Tyrrhenian marine sample (DED8708), and Greek lacustrine sample (TP), excludes a post-depositional origin. It is reasonable to assume that the cristobalite occurring is neither “vapor-phase cristobalite”^76^, nor syn-eruptive cristobalite^77^, as also confirmed by the exclusive presence of the alpha-cristobalite. The most reliable hypothesis is that cristobalite is generated by hydrothermal alteration of volcanic edifice rocks^78^ and then incorporated to the ash cloud during the eruption producesses.

Our results highlight the complexities involved in the dispersal of volcanic ash, particularly of fine ash fractions, over long distances. In particular, while on one hand we observed an expected progressive decrease in coarser ash fractions and the relative increase in finer fractions with increasing distance from the source (Supplementary Fig. S4), which coincides with the fact that larger particles tend to settle closer to the vent due to their higher terminal velocities, on the other, relatively coarse particles (> 63 µm) are still present at great distances (e.g., DRA-1). This is because the distal samples are a mix between the two eruptive phases and probably the coarser fraction belongs to the Plinian phase, which is coarser than the co-ignimbritic phase^70^.

Focusing on the fine fraction (< 63 µm), the maximum size of the particles in the distal samples decreases with distance from the source (Fig. 2), with the coarser glass (up to 57 µm) in the DED 8708 sample (Tyrrhenian Sea) than in the TP (Greece) and DRA-1 (Romania) samples (up to 44 µm and 43 µm, respectively), similarly to the mineral phases (feldspar and SiO_2_). This confirms the previous observation that larger particles settled before finer ones^11,16,79^. The latter can cover a wider area due to their lower terminal velocities, as also observed in the results obtained on *v*_*t*_ (Fig. 4). This finding agrees with past studies that emphasized the role of particle size in controlling the settling velocity and residence time of ash in the atmosphere^11,16,79^. In addition, our results show that transport and dispersion dynamics are also influenced by the density and the shape of ash particles. Indeed, this study shows that for all the samples the mineral phases are concentrated in finer fractions and their amount decreases with increasing distance from the source, in contrast to glass (Fig. 2, Supplementary Fig. S3), which agrees with the results obtained by Mele et al.^80^ on other Campi Flegrei samples, and Cashman and Rust^81^ observations on Quizapu and Mount St. Helens eruptions. The prevalence of glass in coarser ash fractions, particularly in the ultra-distal samples (TP and DRA-1), is consistent with the fact that glass particles have a lower *v*_*t*_ and are more easily transported over long distances compared to crystalline phases, due to their lower density (*ρ*_*glass*_ = 2500 kg/m^3^, *ρ*_*feldspar*_ = 2560 kg/m^3^, *ρ*_*SiO2*_ = 2650 kg/m^3^) and more flattened and elongated shape (Fig. 3, Table 2). The latter is more irregular for glass particles than the mineral phases, which tend to be more compact and denser (Fig. 3, Table 2) and thus settle faster for their different aerodynamic behaviours, as evidenced by mineral phases *v*_*t*_, which is slightly higher than the glass one (*v*_*t*, feldspar/_*v*_*t*, glass_ = 1.05 ± 0.03, *v*_*t*, SiO2/_*v*_*t*, glass_ = 1.09 ± 0.02). The higher *v*_*t*_ of both feldspars and crystalline SiO₂ is due to a combination of shape and density effects. When considering the influences of these two factors separately, both contribute to an increase in *v*_*t*_ as follows: the more compact shape had a similar impact on feldspars and crystalline SiO_2_ *v*_*t*_ (*v*_*t, S feldspar*_*/v*_*t,*__*S glass*_ = 1.03 ± 0.2, *v*_*t, S SiO2*_*/v*_*t,*__*S glass*_ = 1.03 ± 0.2, Supplementary Fig. S5b,c) and its contribution was similar to that of density for feldspars (*v*_*t, ρ feldspar*_*/v*_*t,*__*ρ glass*_ = 1.02 ± 0.002) whereas for crystalline SiO₂ density plays the dominant role (*v*_*t, ρ SiO2*_*/v*_*t,*__*ρ glass*_ = 1.06 ± 0.001 Supplementary Fig. 5a, c). This trend is reflected also in the distribution of SiO₂ grains, which are almost absent in the most distal samples and more abundant in the proximal ones. Similarly, feldspar content decreases with increasing distance from the source (Supplementary Fig. S3).

The results of this study confirm the overestimation of terminal velocity by spherical particle approximation models, particularly for the glass component (Fig. 4b), indicating that the spherical approximation is inadequate for accurate predictions of ash settling behaviour^11,13^. Indeed, this overestimation could lead to underestimating the atmospheric residence time and fallout time of the ash. This result emphasizes the importance of using shape-specific models for the calculation of ash dispersal and deposition, particularly when considering fine ash particles that may be transported globally^16^.

One of the most significant findings of our study is the low terminal fall velocity (*v*_*t*_) of the fine ash particles (< 63 µm), in the order of a few cm/s (from 0.01 to 17.03 cm/s) (Fig. 4a). This aspect is of paramount importance for the < 10 µm particles, particularly those in the respirable range (< 4 µm), which are known to pose the greatest health hazard^7^. The calculated terminal velocities for all the samples are < 0.8 cm/s and < 0.1 cm/s for < 10 µm (PM10) and < 4 µm (PM4) particles, respectively, which are extremely low values supporting the prolonged atmospheric residence times of these particles. Therefore, very fine volcanic ash can have a very slow fallout rate, remaining in the atmosphere for days or even weeks, as also pointed out by Rose and Durant^16^. It can contribute to long-range transport with fine ash covering thousands of kilometres from the vent, as demonstrated by the distal samples (TP and DRA-1 at 848 km and 940 km, respectively). It is important to consider that in this study the effect of fine ash (< 63 μm) particle aggregation, which could lead to premature sedimentation^82,83^, is not considered. Indeed, the focus of this work is on investigating the aerodynamic behaviour of each particle individually.

Building on the foundation established by previous studies on the influence of shape on *v*_*t*_ [i.e.^10,11^] and on aerodynamic characteristics of volcanic ash particles [i.e.^80,81^], this study provides an innovative perspective by determining, for the first time, the effect of both shape and density on v_t_ for each component of volcanic ash, distinguishing between mineral phases and glass. Notably, the detailed study of particle shape and its consequences on *v*_*t*_ was performed here for the first time on a super-eruption, and specifically on respirable fraction.

In particular, the present work provides numerical data based on the real aerodynamic properties of fine ash particles from super-eruption, laying the foundation for improving numerical dispersal models and representing the starting point to better understand the behaviour of ash particles.

## Implications for health hazards

The hypothesis that cristobalite may derive from altered rocks of volcanic edifice involved in the eruption opens an interesting avenue on the health hazard issue. Indeed, it raises the possibility that even SiO_2_ non-supersaturated eruptions could contain cristobalite in their products, which can be dispersed over long distances, thus representing a potential health hazard.

One of the key implications of this study is the identification of the < 10 μm fraction, known as PM10, and the < 4 μm fraction, which poses a potential health hazard due to its ability to penetrate deeply into the respiratory system, reaching the alveoli^7^. The fractionation and transport of fine ash are critical in assessing the potential for long-term health impacts in areas affected by volcanic ash deposition, even far from the eruption source. Indeed, the < 4 μm and 5–8 μm fractions, which correspond to the respirable and thoracic fractions respectively, are present in all CI distal samples (up to 940 km) due to their long-distance transport and their low *v*_*t*_ (< 0.1 and < 0.5 cm/s respectively), proving that they can affect very wide areas including areas located near the volcanic eruption source (e.g., DED8708). Given the widespread dispersal of ash from the CI super-eruption, even populations hundreds of kilometres away from the eruption may be exposed to fine ash inhalation as a result of a super-eruption. Co-ignimbrite plumes for example dispersed ash up to 3200 km away from the vent^60,64^ over an area of ~ 3 million km^2^^59^. Another critical aspect to consider is the long-term environmental and health impact of volcanic fine ash, which is exacerbated by its ability to be resuspended by wind and anthropogenic activities, especially in dry and windy environments^8^. The threshold friction velocity for ash resuspension is generally low, approximately 0.4 m/s, assumed regardless of ash size and environmental conditions^84^, suggesting that resuspension events can occur frequently in regions where fine ash is deposited on unconsolidated surfaces by even moderate winds. Considering that particles < 20 µm can be remobilized into long-term suspension (weeks-months)^85^, on that front, the CI provides an opportunity to assess whether fine ash, which can be subjected to long-time resuspension in middle, distal and ultra-distal locations (DED 8708, TP and DRA-1), can lead to potential health hazard from prolonged inhalation.

Our findings therefore enhance the understanding of volcanic ash aerodynamic behaviour and offer new perspectives on the extensive impacts of super-eruptions, highlighting associated health hazards.

## Materials and methods

### Materials

Samples of the CI eruption from proximal, distal, and ultra-distal areas (i.e., up to 940 km from the source), by considering different depositional environments, were selected (Fig. 1, Table 3). We investigated Santuario subaerial sample from Maddaloni (Santuario San Michele e Santa Maria del Monte, Caserta, Italy), DED 8708 marine sample from Tyrrhenian Sea (Italy)^86,87^, TP sample from the Tenaghi Philippon peat bog (Greece)^88–90^, and DRA-1 sample from the Draganesti-Olt loess-paleosol profile (Romania)^91^.

**Table 3:** Overview of locations, depositional environments, and age of CI samples analysed in this study. All the information reported in DED 8708, TP, and DRA-1 samples are from references listed.

Sample	Lat	Long	Distance from source (km)	Thickness (cm)	Depositional environment	Dating method	Age (ka)	Correlation references
Santuario	41.05° N	14.40° E	33	–	Subaerial	^40^Ar/^39^Ar	39.8 ± 0.4	This work
DED 8708	39.42° N	13.35° N-	200	~ 35	Marine	^40^Ar/^39^Ar	41.1 ± 2.1	Thon-That et al. (2001) and Paterne et al.^87^
TP	40.97° N	24.22° E	848	~ 25	Lacustrine	Radiocarbon	39.7 ± 0.1	Staff et al.^90^
DRA-1	44.16° N	24.55° E	940	~ 60–75	Loess	Luminescence on loess	40.4 ± 1.3	Veres et al.^91^

Santuario sample is localized and correlated for the first time in this study.

Except for the Santuario unit, based on robust chronological constraints and geochemical fingerprinting, all the analysed distal tephra were previously confidentially attributed to the CI. Therefore, in order to make stronger the attribution of the Santuario pumice fall to the CI Plinian phase, a sample from this unit was dated by ^40^Ar/^39^Ar method (see details in Supplementary Table S1 and S2).

Considering stratigraphy and chemistry, these samples are interpreted as primary deposits. The Santuario sample belongs to the Plinian fall-out phase and consists of ~ 60 cm-thick fall deposit made of lapilli-sized (2–64 mm) high-vesicular and angular grey pumices.

The DED 8708 sample comes from the top of the ash layer, located 200 km from the source (Table 3). Given that within 600 km from the vent, the co-ignimbrite tephra overlaps the Plinian phase, and that within 850 km approximately 60 ± 6% of the volume originates from the co-PDC phase^70^, this sample is consistent with the co-ignimbrite phase.

The distal and ultra-distal samples TP and DRA-1 are from the base of the ash layers and they likely correspond to both phases, Plinian and co-PDC, mixed^70^, both are predominantly associated with the Plinian and early LPF co-PDC phases (Fig. 1a), having ~ 35% of UPFU co-ignimbritic tephra^60^.

## Methods

### ^40^Ar/^39^Ar dating

The ^40^Ar/^39^Ar age of Santuario sample was obtained at the Laboratoire des Sciences du Climat et de l’Environnement (CEA, CNRS UMR 8212, Gif-sur-Yvette, France) dating facility, through the following steps:

1. Fresh and transparent K-rich feldspars were extracted from the Santuario sample.
2. After being washed in distilled water, transparent K-feldspars (500 µm–1 mm) without any visible inclusions were handpicked under a binocular and used for dating.
3. Before irradiation, these crystals were leached in a 10% HF solution for 5 min to remove any potential groundmass fragments and alteration phases that might still be attached to the surface of the crystals.
4. Approximately 30 individual crystals were irradiated in the Cd-lined, in core CLICIT facility of the Oregon State University TRIGA reactor for 2 h (IRR. CO-013). Interference corrections were based on the nucleogenic production ratios given by Balbas et al.^92^.
5. After irradiation, 17 individual crystals were transferred into a copper 133 pits sample holder placed into a differential vacuum Teledyne Cetac window connected to a home-designed compact extraction line.
6. Minerals were fused one by one using a 100 W Teledyne Cetac CO_2_ laser for 20 s at 2.5 W. Before fusion, each crystal underwent a 20 s long sweeping at 0.3 W to remove unwanted gas potentially trapped on the crystals surface and fractures.
7. Extracted gases were firstly purified by a SAES GP 50 cold getter for 90 s and then for 230 s by two hot SAES GP 50 getters. The five Argon isotopes (i.e., ^40^Ar, ^39^Ar, ^38^Ar, ^37^Ar, and ^36^Ar) were measured using a multi-collector NGX 600 mass spectrometer equipped with 9 ATONA^®^ amplifiers array and an electron multiplier. More technical specifications regarding the NGX 600 ATONA detector array are presented in detail by Cox et al.^93^.
8. ^40^Ar, ^39^Ar, ^38^Ar, and ^36^Ar isotopes were collected simultaneously while the ^37^Ar was measured in a second time. In the first run, ^40^Ar, ^39^Ar, and ^38^Ar were measured simultaneously on 3 ATONA^®^ amplifiers and ^36^Ar on the electron multiplier. Following this first run, the ^37^Ar was measured alone using the electron multiplier.

Each isotope measurement corresponds to 15 cycles of 20 s integration time. Peak intensity data were reduced using ArArCALC V2.4^94^. Neutron fluence J factor was calculated using co-irradiated Alder Creek sanidine standard ACs-2 associated to an age of 1.1891 Ma^95^ according to the K total decay constant of Renne et al.^96^ (λ_e.c._ = (5.757 ± 0.016) × 10^ −11^ yr^−1^ and $\lambda _{{\beta ^{ - } }}$ = (4.9548 ± 0.0134) × 10 ^−10^ yr^−1^)). To determine this neutron flux we used at least 6 flux monitor crystals coming from pits framing the sample in the irradiation disk. J-value obtained is 0.00055240 ± 0.00000033. To verify the detectors linearity, mass discrimination was monitored by analysis of at least 50 air shots of various beam sizes ranging from 1.0 × 10^−2^ up to 5.0 × 10^−2^ V (1–5 air shots). About 15 air shots analyses were performed every day. These measurements were done automatically during the nights before and after the unknown measurements. Discrimination was calculated according to the ^40^Ar/^36^Ar ratio of 298.56^97^. Procedural blank measurements were achieved after every two to three unknowns. For typical 5 min time blank, backgrounds are between 2.2 and 3.5 × 10^ −4^ V for ^40^Ar and 80−100 cps for ^36^Ar (about 1.0 × 10^−6^ V equivalent). Weighted mean age uncertainties are reported at 2, including J uncertainty, and were calculated using Isoplot 4.0^98^. Full analytical data for each sample can be found in Supplementary Table S1 and S2.

### Particle size separation protocol

The middle-distal, and ultra-distal samples (DED 8708, TP, and DRA-1) were prepared with the same methodology, whereas the proximal sample (Santuario) was subjected to a different procedure.

Concerning the latter, pumice lapilli from the base, middle, and top of the deposit were chosen and each one was sectioned and embedded in epoxy resin to be analyzed by SEM at ‘Rock crushing and thin section laboratory’ of Sapienza University of Rome.

On the distal samples, a particle size fractions separation protocol was applied instead. The size separations were performed by following steps:

1. Samples were mixed in 500 ml of distilled water and 50 ml of sodium hexametaphosphate (NaPO_3_)_6_ with a concentration of 20 g/l with dispersant function.
2. Mechanical rod stirring for 3 h.
3. Separation of the > 63 μm fraction by wet sieving.
4. Fine ash with a < 63 μm size remaining in distilled water and dispersant was introduced in a settling cylinder; water was added up to 1 L.
5. Separation by decantation was carried out by picking up the sediment suspended above the cylinder tap following Bellotti and Valeri^99^
6. After each collection, 500 ml of distilled water and 25 ml of dispersant were added to restore the appropriate experimental conditions.

Collections were performed to separate the < 4 μm fraction, relevant for lung toxicity, 5–8 μm, 9–16 μm, and 17–63 μm fractions.

The particle size separation protocol was performed at ‘Sedimentology laboratory’ of Sapienza University of Rome. To ensure the effectiveness of the particle size division protocol, the fractions obtained were observed by SEM and the equivalent diameter of at least 70 particles per fraction was calculated by measuring their dimensions with ImageJ software (https://imagej.net/ij/).

The different particle size fraction (< 4 μm, 5–8 μm, 9–16 μm, 17–63 μm, and 63 μm–mm size) were also weighted in order to determine the grain size distribution of each sample.

### Component analyses

Component analysis was performed through X-Ray Powder Diffraction (XRPD) and Scanning Electron Microscopy (SEM) equipped with energy dispersive X-ray spectroscopy (EDS) at X-ray powder diffraction Laboratory and Scanning Electron Microscope Laboratory of La Sapienza di Roma, respectively. XRPD analyses were performed on bulk samples without particle size separation for a qualitative study. X-ray powder diffraction (XRPD) analyses of the samples to identify mineral phases were performed using a Bruker AXS D8-Advance diffractometer, operating in θ/θ transmission mode, equipped with incident-beam focusing Gobel mirrors and a PSD VÅntec-1 detector. The powdered samples were loaded into 0.6 mm diameter borosilicate capillaries. Data were collected using CuKα radiation (λ = 1.5418 Å, 40 kV at 40 mA), in the 5–145°2θ angular range, with a step size of 0.022°2θ and 3 s counting time.

Components quantification was carried out by SEM–EDS observations, using a FEI Quanta 400 equipped with an EDAX Genesis microanalysis system. Modal analysis was carried out on top, middle, and base pumices of Santuario sample, by counting the element occurring at each node of a mesh with 400 μm spacing, over an area of ~ 90 mm^2^ on a surface section of each pumice. A general quantification of glass, vesicles (i.e., void fraction), and crystals in the top, middle, and base sub-samples was performed, determining their area percentages within the pumices through Scanning Electron Microscopy (SEM) observation.

On the middle, distal, and ultra-distal samples (DED 8708, TP, and DRA-1), the grain percentage of the main phases (glass, feldspar, and crystalline SiO_2_) were determined for specific size fractions: < 4 μm, 5–8 μm and 9–63 μm, through grain counting under SEM, focusing exclusively on the fine fractions excluding the coarser fraction (i.e. 63 µm–mm size). In this case, the 9–16 and 17–63 μm fractions were combined to obtain a statistically valid number of grains for quantification (~ 100 grains per fraction).

### Shape characterization

We determined the shape of particles from DED 8708, TP, and DRA-1 for each size fraction, 4 μm, 5–8 μm, 9–16 μm, and 17–63 μm (300 grains per samples), differentiating the glass and mineral phases, using secondary electron SEM images processed with the open source image analysis software ImageJ (https://imagej.net/ij/). For each particle, we measured the three *form dimensions*, L: longest, I: intermediate, and S: shortest length of the particle. These particle form factors have a low dependency on the operator error and measurement resolution than sphericity, providing a better discrimination of particles with different forms^43^.

These parameters were measured in the following steps:

1. SEM Images were first converted from RGB to 8-bit and then converted to binary though ImageJ.
2. Using the *Threshold* function, particles were selected. In some cases, due to the poor or non-existent difference between the grey tones of the particles and the background, it was necessary to use a combination of the graphics program Adobe Photoshop, which was employed to manually trace the particles, and the *Threshold* function of Image J software.
3. Assuming the particles are oriented on the stab parallel to their maximum surface, the L: longest (Feret) and I: intermediate (Min Feret) dimensions of the particles were measured using the *Analyze Particles* function of ImageJ, according to the projection area protocol of Bagheri and Bonadonna^10^.
4. In the absence of 3D images, the S: shortest dimension of each particle was instead measured by converting the grey values of the particles into μm, using a proportion obtained by equating the diameter measure of spherical particles to their grey value.

The three *form dimensions* were used to calculate the equivalent diameter, *d*_*eq*_, by averaging them, and *flatness, f,* and *elongation, e, shape descriptors*, defined as^40^:

3 $$f = \frac{S}{I}$$

4 $$e = \frac{I}{L}$$

*Working sphericity, ψ,* was calculated using the formula^43^:

5 $$\psi \approx \frac{{12.8\sqrt[3]{{f^{2} e}}}}{{1 + f\left( {1 + e} \right) + 6\sqrt {1 + f^{2} \left( {1 + e^{2} } \right)} }}$$

The morphology of the particles of each component was evaluated in terms of elongation and flatness, considering the different size fractions (< 4 µm, 5–8 µm, 9–16 µm, and 17–63 µm). Particle shapes were classified according to the classification system proposed by Angelidakis et al.^72^ (Fig. 3).

### Terminal fall velocity calculation

These parameters were used to calculate the terminal fall velocity, *v*_*t*_, for DED 8708, TP, and DRA-1 samples, differentiating between components (glass, feldspar, and crystalline SiO_2_). We applied the Eq. (1) using the drag equation of Bagheri and Bonadonna^10,11^ for nonspherical particles, valid for Re < 3 × 10^5^:

6 $$C_{D} = \frac{{24K_{s} }}{Re}\left[ {1 + 0.125\left( {Re\frac{{K_{n} }}{{K_{s} }}} \right)^{2/3} } \right] + \frac{{0.46k_{n} }}{{1 + \frac{5330}{{Re\frac{{k_{n} }}{{k_{s} }}}}}}$$

where

7 $$k_{S} = 0.5\left( {F_{S}^{\frac{1}{3}} + F_{S}^{{ - \frac{1}{3}}} } \right)$$

and

8 $$k_{N} = 10^{{\alpha 2\left[ { - log\left( {F_{N} } \right)} \right]^{\beta 2} }}$$

F_S_ and F_N_ are defined as:

9 $$F_{S} = fe^{1.3} \left( {\frac{{d_{eq}^{3} }}{LIS}} \right)$$

10 $$F_{N} = f^{2} e\left( {\frac{{d_{eq}^{3} }}{LIS}} \right)$$

Although other equations for *C*_*D*_ calculation were developed (i.e.,^12,31,33^), the Bagheri and Bonadonna^10,11^ model was found to be the most appropriate for our case, considering the size of the particles examined and the data obtainable from 2D images.

Finally, we compared terminal fall velocity values, obtained as above, with velocities calculated by a spherical particle approximation *C*_*D*_ model^26^:

11 $$C_{D} = C_{1} + \frac{24}{{Re}} + \frac{{C_{2} }}{{1 + \sqrt {Re} }}$$

where C_1_ = 0.25 and C_2_ = 6.0 for Re < 5 × 10^3^.

To calculate the Reynolds number, *Re*, necessary in the *C*_*D*_ calculation, we used the iterative procedure indicated by Dioguardi et al.^12^ which consists in calculating *C*_*D*_, *v*_*t*_ and *Re*, starting from a guessed value of *Re*, and then recalculate them until the latest value of *Re* stabilises with those previously obtained.

The *v*_*t*_ was first determined for each component, using their actual properties of shape and density. Then, *v*_*t*_ was determined by separately isolating the effects of shape and density: first, by applying the density of mineral phases while keeping the glass particle shape parameters constant, and then by applying the shape parameters of crystalline phases while keeping the glass density constant.

## Electronic supplementary material

Below is the link to the electronic supplementary material.

Supplementary Material 1

Supplementary Material 2

## Data Availability

The datasets generated during and/or analysed during the current study are available from the corresponding author on reasonable request.
