# Supplementary material for: Fine ash from the Campanian Ignimbrite super-eruption, ~ 40 ka, southern Italy: implications for dispersal mechanisms and health hazard
Source: Sci Rep. 2025 Jun 10;15:20039. doi: 10.1038/s41598-025-01100-4 (PMC12152150; doi:10.1038/s41598-025-01100-4)
Supplement: Supplementary file 1 — Supplementary Material 1 [file 41598_2025_1100_MOESM1_ESM.pdf]

Supplementary information for

Fine ash from the Campanian Ignimbrite super-eruption, ~40 ka, southern Italy: implications for dispersal mechanisms and health hazard

Flaminia Gianchiglia*, Paolo Ballirano, Biagio Giaccio, Andreas Koutsodendris, Sebastien Nomade, Alessandro Pacella, Danilo M. Palladino, Jörg Pross, Daniel Veres, Gianluca Sottili.

*Flaminia Gianchiglia
Email: flaminia.gianchiglia@uniroma1.it

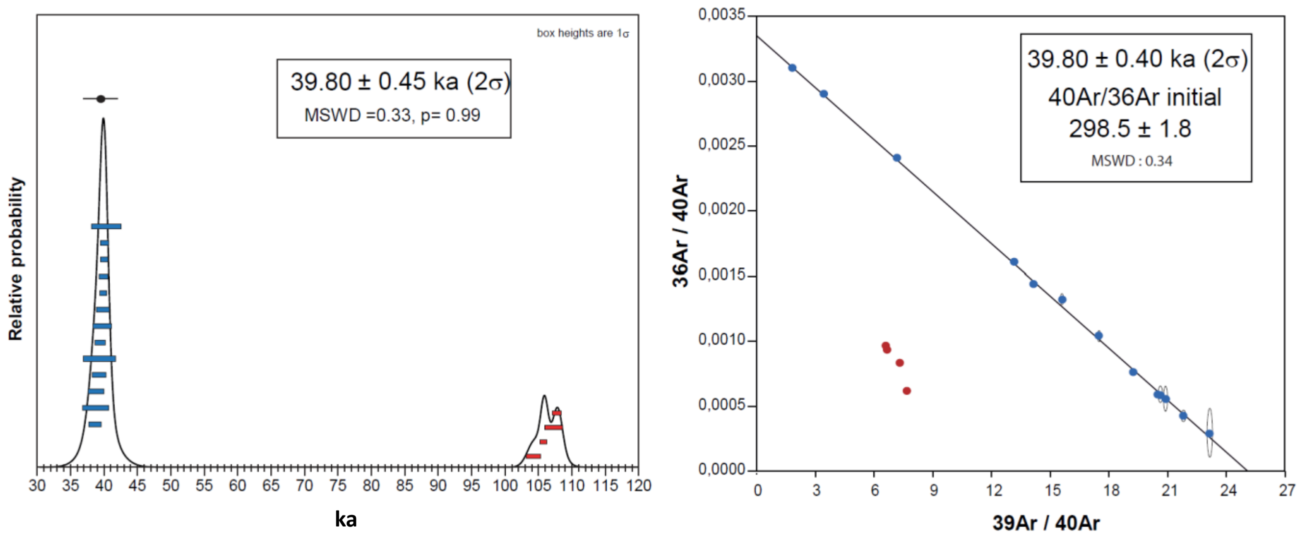

Supplementary Figure S1. Probability diagrams representing $^{40}Ar/^{39}Ar$ dating result of Santuario sample. The inverse isochron, corresponding to the youngest population of crystals, have a spreading of 84.9 % with an initial intercept of 298.5 ± 0.9 (1s), therefore coherent with an atmospheric value suggesting that these crystals do not contain any excess argon.

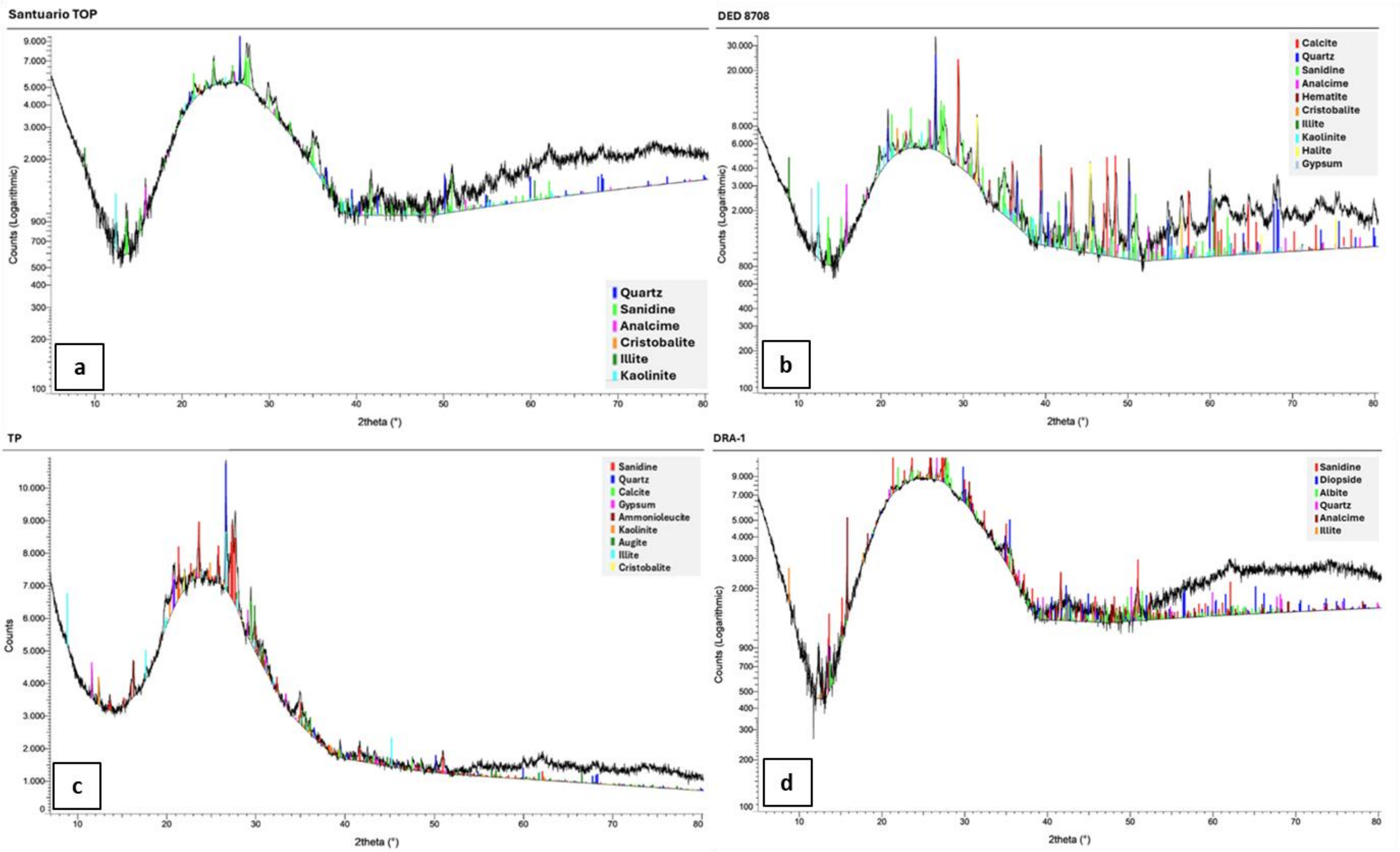

Supplementary Figure S2. XRD spectra of (a) Santuario pumice, (b) DED 8708, (c) TP and (d) DRA-1 ash samples. Coloured vertical bars refer to the position of the Bragg reflections of the identified mineral species. Colour code reported as inset.

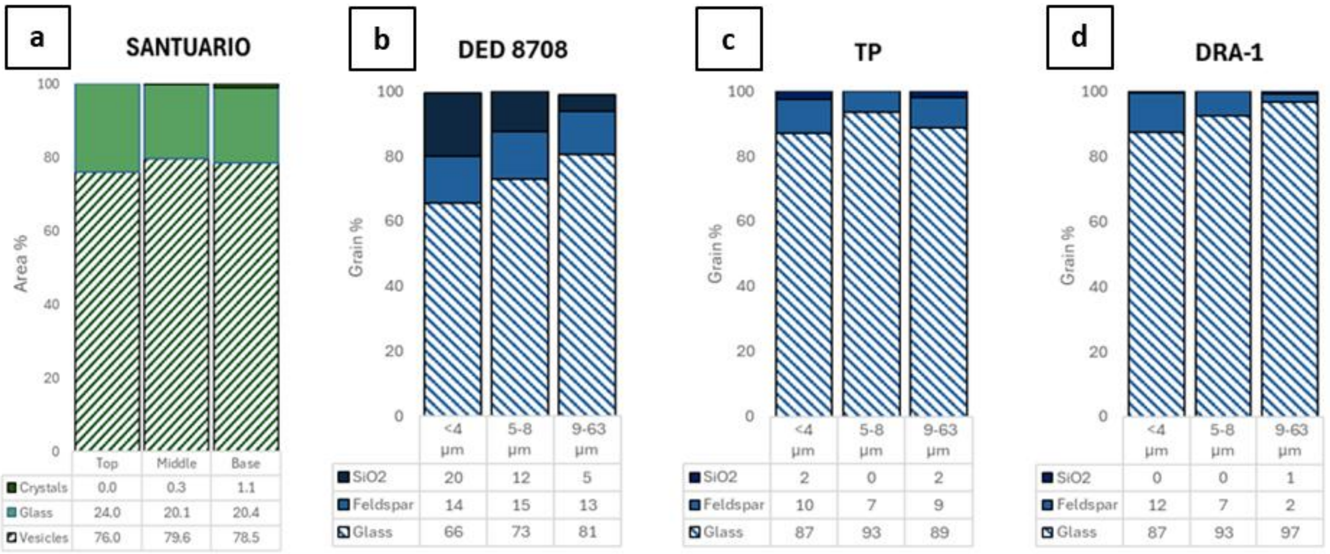

Supplementary Figure S3. (a) Area% of glass, vesicles, and crystals in Santuario sample from top, middle and base. Grain% of glass, feldspar, and SiO_2 across different size fractions, in DED 8708 (b), TP (c) and DRA-1 (d) samples.

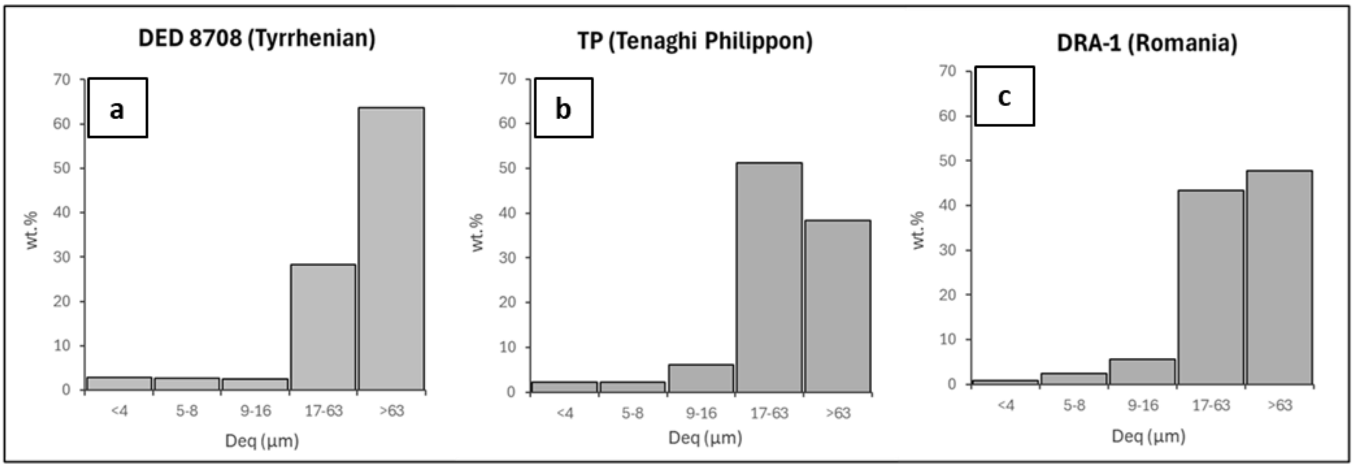

Supplementary Figure S4. (a) Area% of glass, vesicles, and crystals in Santuario sample from top, middle and base. Grain% of glass, feldspar, and SiO_2 across different size fractions, in DED 8708 (b), TP (c) and DRA-1 (d) samples.

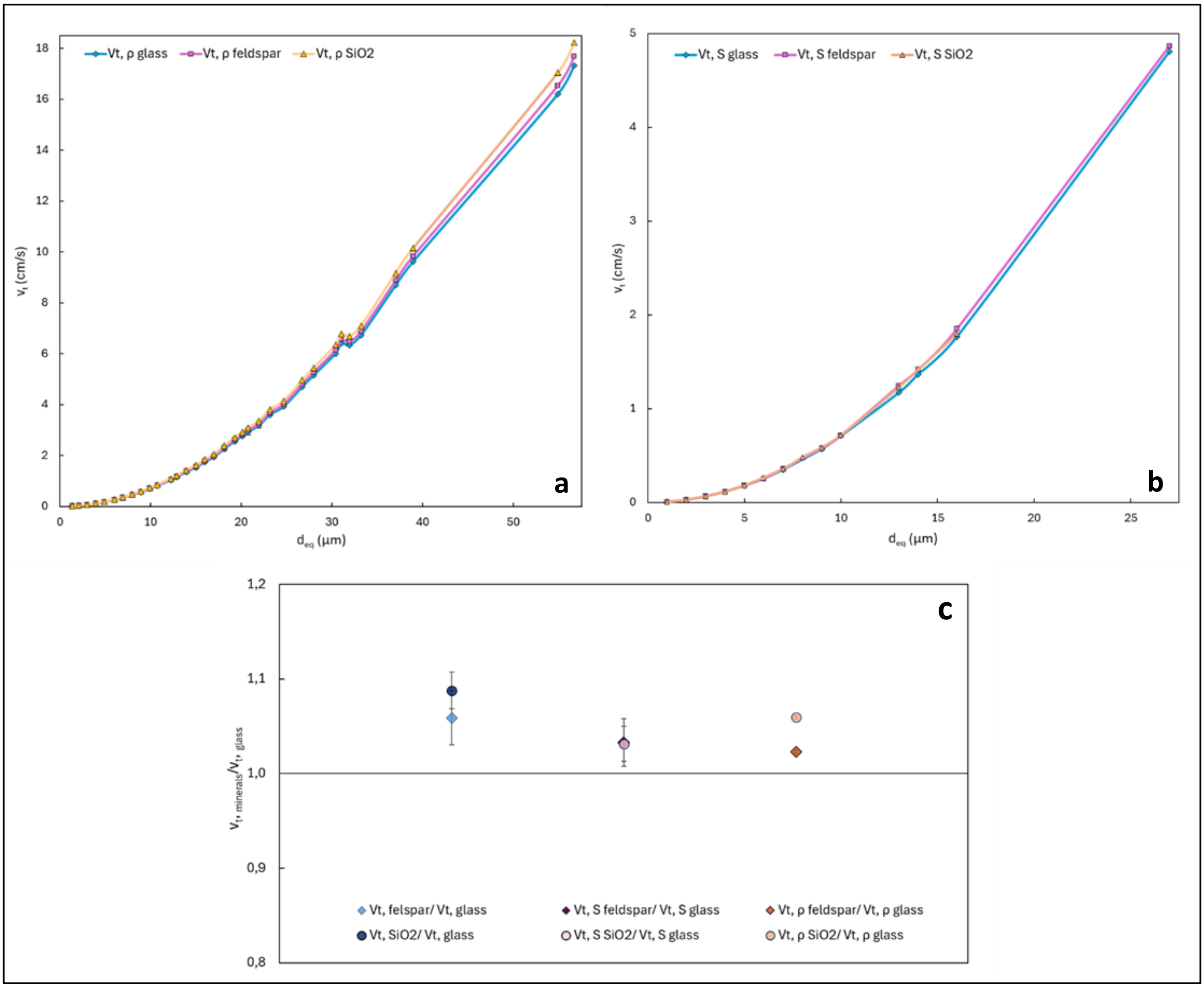

Supplementary Figure S5. Comparison of v_t calculated for (a) the different densities of glass, feldspar, and SiO_2 (ρ_{glass} = 2500 kg/m^3, $\rho_{feldspar}$ = 2560 kg/m^3, ρ_{SiO2} = 2650 kg/m^3) keeping the glass particle shape parameters constant; (b) the different shape descriptors of each component, with glass density constant. (c) Comparison of v_t ratio of v_t of the mineral phases and glass ($v_{t,\ feldspar}/\ v_{t,\ glass}$, $v_{t,\ sio2}/\ v_{t,\ glass}$), v_t accounting only the shapes of components ($v_{t,\ s\ feldspar}/\ v_{t,\ s\ glass}$, $v_{t,\ s\ sio2}/\ v_{t,\ glass}$), and v_t accounting only the densities of the components ($v_{t,\ \rho\ feldspar}/\ v_{t,\ \rho\ glass}$, $v_{t,\rho\ sio2}/\ v_{t,\ \rho\ glass}$).
